# FGF Family: From Drug Development to Clinical Application

**DOI:** 10.3390/ijms19071875

**Published:** 2018-06-26

**Authors:** Qi Hui, Zi Jin, Xiaokun Li, Changxiao Liu, Xiaojie Wang

**Affiliations:** 1 School of Pharmacy, Wenzhou Medical University, Chashan University Park, Wenzhou 325035, China; huiqi1976@163.com (Q.H.); 18858818119@163.com (Z.J.); lixk1964@163.com (X.L.); liuchangxiao@163.com (C.L.); 2 Key Laboratory Biotechnology Pharmaceutical Engineering, Wenzhou Medical University, Chashan University Park, Wenzhou 325035, China; 3 State Key Laboratory of Drug Delivery Technology and Pharmacokinetics, Tianjin Institute of Pharmaceutical Research, 308 Anshan West Road, Tianjin 300193, China

**Keywords:** fibroblast growth factor, wound healing, metabolic regulation, fibroblast growth factor receptor inhibitor, drug development, clinical application

## Abstract

Fibroblast growth factor (FGF) belongs to a large family of growth factors. FGFs use paracrine or endocrine signaling to mediate a myriad of biological and pathophysiological process, including angiogenesis, wound healing, embryonic development, and metabolism regulation. FGF drugs for the treatment of burn and ulcer wounds are now available. The recent discovery of the crucial roles of the endocrine-acting FGF19 subfamily in maintaining homeostasis of bile acid, glucose, and phosphate further extended the activity profile of this family. Here, the applications of recombinant FGFs for the treatment of wounds, diabetes, hypophosphatemia, the development of FGF receptor inhibitors as anti-neoplastic drugs, and the achievements of basic research and applications of FGFs in China are reviewed.

## 1. Introduction

The first Fibroblast growth factor (FGF), also known as bFGF or FGF2, was identified in pituitary extracts by Armelin in 1973 [1] and then was also found in a cow brain extract as a component that caused fibroblasts to proliferate [2]. FGFs belong to a large family of growth factors that includes 23 family members [3]. FGF family members share sequence and structural similarity [4]. Through paracrine or endocrine secretion, FGFs participate in important pathophysiological processes such as angiogenesis, wound healing, embryonic development, and endocrine secretion regulation [5,6,7,8,9]. The diverse biological functions of FGFs depend on their binding to the cofactor heparan sulfate (HS) or Klotho to form dimers with their receptors (FGFRs) [10,11,12]. Because of their extensive functions, FGF family members and their receptors are important targets for drug development. Many studies have reported the participation of FGF signaling in the regulation of multiple processes in embryonic development, including zygote implantation, gastrulation, morphology development, and organ formation [13,14]. Recent clinical and biological studies demonstrated that FGF signaling mediates metabolic functions including maintenance of phosphate/vitamin D balance and cholesterol/bile acid balance, and regulation of glucose/lipid metabolism [15,16]. These diverse biological functions may make FGF signaling beneficial in the treatment of many human diseases including congenital craniosynostosis, dwarf syndrome, Kallmann syndrome, hearing loss, hypophosphatemia, and various types of cancers [14,17]. Endocrine FGF subfamily members could be useful for the treatment of chronic kidney disease, obesity, and insulin resistance [13]. Over the past decade, important findings of the structure and molecular mechanism of FGFs changed our understanding of FGF signals in human health and disease occurrence and development, stimulating new strategies for drug development with FGF and its receptors as targets. This review summarizes the physiological and pathological roles of FGF and recent research and application advances, to stimulate continued efforts in FGF drug development.

## 2. FGFs and Their Receptors

FGFs and their receptors (FGFRs) play essential roles to tightly regulate cell proliferation, survival, migration, and differentiation during development and adult life. Classification of FGF family members and the known structural characteristics of FGFs and FGFRs are presented here.

### 2.1. Classification of FGF Family Members

**Figure 1:**
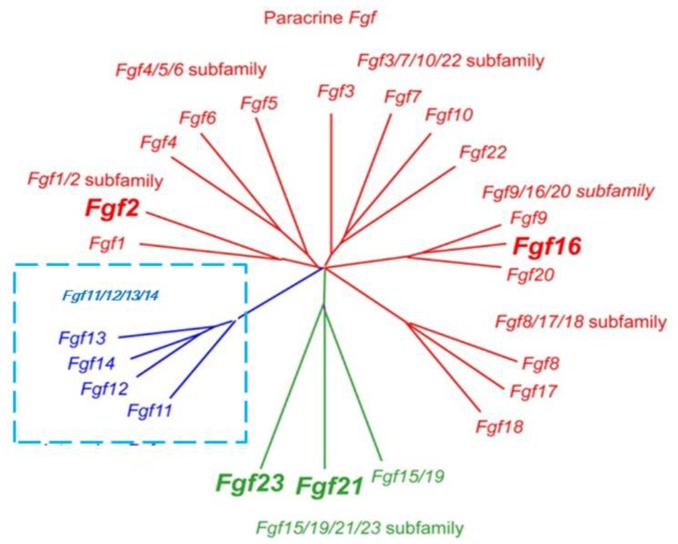
Evolutionary relationships within the human/mouse *Fgf* gene family by phylogenetic analysis. Phylogenetic analysis suggests that 18 mammalian FGFs are classified into six subfamilies containing five paracrine subfamilies and an endocrine subfamily. FGF members such as FGF11-FGF14 are not classified into the above six subfamilies because they cannot activate FGF receptors.

Based on sequence homology and developmental characteristics, 18 mammalian FGFs are classified into six subfamilies: Five paracrine subfamilies and an endocrine subfamily [14]. The five paracrine subfamilies are FGF1 subfamily (FGF1 and FGF2), FGF4 subfamily (FGF4, FGF5, and FGF6), FGF7 subfamily (FGF3, FGF10, FGF7, and FGF22), FGF8 subfamily (FGF8, FGF17, and FGF18), and FGF9 subfamily (FGF9, FGF16, and FGF20) (Figure 1). Classic FGF family members are considered paracrine factors that regulate the formation of tissue and organs during embryonic development. In contrast, the FGF19 subfamily members (FGF19, FGF21, and FGF23) are endocrine FGFs that act through endocrine secretion. Other FGF members such as FGF11-FGF14 are not classified into the above six subfamilies. Although they are highly homologous to the other FGF family members, they do activate FGF receptors [18,19]. Endocrine FGF19 subfamily members regulate the balance of bile acid, cholesterol, glucose, vitamin D, and phosphate by binding to the specific tissue-dependent Klotho protein [5,7,14,20].

### 2.2. Structural Characteristics of FGF Family Members and Their Receptors

**Figure 2:**
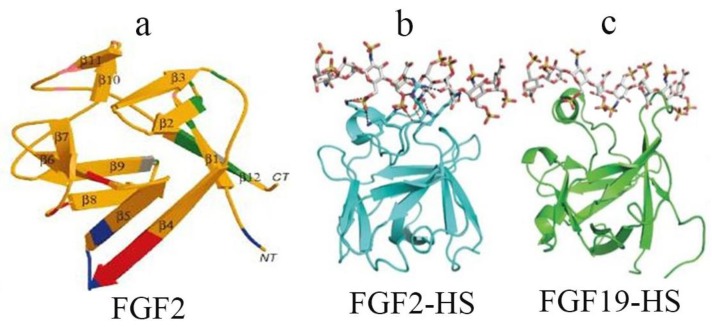
Comparison of the crystal structures of FGF2 and FGF19 provides the structural basis for the low affinity of endocrine ligands for HS. (a) Three-dimensional structure of FGF2, a prototypical member of the FGF family. A ribbon diagram of FGF2 is shown; β strands are labeled 1–12. The heparin-binding region (pink) includes residues in the loop between β strands 1 and 2 and in β strands 10 and 11. Residues that contacted the FGFR are shown in green (the region contacting Ig-domain 2 of the receptor), blue (contacting Ig-domain 3) and red (contacting the alternatively spliced region of Ig-domain 3); (b) the crystal structures of FGF2 and heprin; (c) the crystal structures of FGF19 and heprin.

FGF family members share about 25–50% sequence homology including a core axis region comprised of 120–130 amino acids that are folded into 12 reverse parallel chains and form a cylindrical structure [4,14,18,21]. All FGF family members, except FGF1, FGF2, FGF9, FGF16, and FGF20, include a signal peptide that targets FGFs for secretion through the classic endoplasmic reticulum-Golgi complex secretory pathway. In contrast, the secretion of FGF9, FGF16, and FGF20 occurs through endoplasm reticulum-Golgi complex-independent pathways. FGFs are heparin sulfate-dependent proteins. The HS-binding site of FGFs is defined by a β1–β2 loop structure and an extended β10–β12 region, presenting solvent-exposed basic amino acids and backbone atoms for HS binding. In the paracrine FGFs, the binding of heparan sulfate results in the FGF ligand forming a continuous surface with positive charges. Differently, in the endocrine FGF subfamily members that do not use HS as a cofactor (FGF19, FGF21, and FGF23), the protein β1–β2 loop and β10–β12 protein elements of the heparan sulfate (HS) binding site instead form a bridge that reduces the binding of FGF and heparan sulfate [14] (Figure 2).

**Figure 3:**
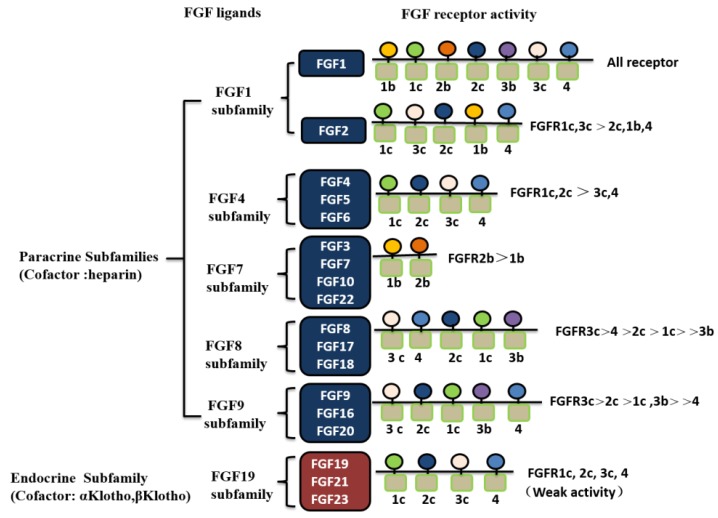
The family of FGF ligands and FGF receptors. The FGF1, FGF4, FGF7, FGF8, and FGF9 subfamily genes encode paracrine FGFs, which bind to and activate FGFRs with heparin/HS as a cofactor. The FGF19 subfamily members encode endocrine FGFs, which bind to and activate FGFRs with the Klotho family protein as a cofactor. The FGF receptors gene family is comprised of four members, FGFR1-FGFR4. Among them, FGFR1–FGFR3 generate two major splice variants of immunoglobulin-like domain III, referred to as IIIb and IIIc, which are essential determinants of ligand-binding specificity.

Fibroblast growth factor receptors (FGFRs) are transmembrane tyrosine kinase receptors that mediate FGF signal transduction through either heparan sulfate- or Klotho-dependent pathways [22]. Four types of independent gene-encoded FGFRs have been identified: FGFR1, FGFR2, FGFR3, and FGFR4. These FGFRs are single-chained glycoproteins, consisting of an extracellular region, a transmembrane region, and an intracellular region. The extracellular region of FGFRs contains three immunoglobulin-like domains (D1–D3) with a serine-rich acid box between the D1 and D2 domains. The transmembrane region is a transmembrane helix, and the intracellular region is the tyrosine kinase region. FGFRs have three common characteristics: (1) Overlapping recognition and multi-specificity, so that one FGFR receptor is capable of binding to different FGFs with similar binding affinity; similarly, each FGF is capable of binding to different FGFRs [23]; (2) The binding of FGFs to their receptors is either cell surface heparan sulfate- or Klotho-dependent [24]; (3) The same gene can produce multiple isoforms of cell-binding and secreting receptors that are formed by the alternative splicing of mRNA during transcription or produced by enzymatic hydrolysis on the cell surface [25]. FGFRs may regulate FGF affinity by competing with other surface receptors for ligand binding. The biological activity of FGFs is mediated by binding to FGFRs to initiate intracellular signal transduction, leading to receptor activation and autophosphorylation of the tyrosine receptor. To date, eight crystal structures of the FGF ligand-bound form of the intracellular kinase region and extracellular D2-D3 region of FGFRs have been resolved: FGF1-FGFR1c, FGF1-FGFR2c, FGF1-FGFR3c, FGF1-FGFR2b, FGF2-FGFR1c, FGF2-FGFR2c, FGF8b-FGFR2c, and FGF10-FGFR2b [14]. NMR analysis of the structure of the D2-D3 region of FGFRs shows that D3 is essentially a flexible region [26,27]. However, X-ray structural analysis of the FGF ligand-binding region reveals that D3 is folded into a stable immunoglobulin-like structure [28]. The FGF-FGFR complexes regulate cell proliferation, differentiation, and migration through complicated signal transduction pathways in various tissues [29,30,31,32]. The relationships of FGF receptors and FGF ligands are shown in Figure 3.

## 3. Drug Development and Clinical Application of FGF Family

Advances in our understanding of the structure and molecular mechanisms of FGF have provided opportunities for new drug development. In the next section, applications of recombinant FGFs for the treatment of wounds, diabetes, hypophosphatemia, the development of FGF receptor inhibitors as anti-neoplastic drugs, and the progress in basic research and successful FGF applications in China are reviewed.

### 3.1. New FGF Drugs for Wound Healing and Their Clinical Application

FGF has been recommended by the US Wound Healing Society and European Wound Management Association for the treatment of refractory ulcers. Since the early 1980s, world-renowned pharmaceutical companies such as Amgen, Merck, and Lilly have significantly invested in the development of FGF-based drugs. Within the FGF family, FGF2, FGF-7 (or keratinocyte growth factor 1, KGF-1), and FGF-10 (or keratinocyte growth factor 2, KGF-2) have been shown to be integral in cutaneous wound healing [33].

In 2004, Amgen released an intravenous formulation of FGF7 (palifermin) that was approved by the US Food and Drug Administration under the trade name Kepivance for use in the treatment of mucositis caused by chemotherapy in leukemia patients with bone marrow transplantation. Clinical studies of palifermin included 212 patients who received high-doses of chemotherapy and radiotherapy for leukemia or lymphoma. Patients received intravenous infusion of palifermin three days before and three days after the aforementioned anti-cancer therapy. Results showed that the palifermin group significantly improved mucositis and shortened the duration compared with the placebo group [34,35,36].

FGF10 (repifermin) has been tested clinically with mixed results. A randomized, double-blind, parallel-group, placebo-controlled, multicenter, 12-week study was conducted to evaluate the safety and efficacy of topical repifermin treatment for the healing of chronic venous leg ulcers [37]. Repifermin accelerated wound healing, as significantly more patients achieved 75% wound closure in the treatment group compared to the number in the placebo group. However, in another clinical trial conducted by the same group, there was no significant difference in the percentage of venous leg ulcers achieving 100% closure after 20 weeks when administered with topical repifermin [38]. Compared with FGF7 and FGF 10, FGF2 has been used more widely for wound healing, and rh-FGF2 showed potential healing effects for treatment of pressure ulcers, diabetic foot ulcers, and second-degree burns. Topical FGF2 has been approved for use in China and Japan. In 2000, use of rh-FGF2 was approved by the Chinese Food and Drug Administration for the treatment of chronic wounds. In 1988, KAKEN of Japan purchased the recombinant bacteria strain expressing human FGF2 (trafermin) and patents from US’s Johnson & Johnson. After 13 years of research, trafermin was approved in 2001 in Japan for the treatment of pressure sores and skin ulcers (both burn ulcers and foot ulcers) [39].

In one randomized controlled trial, patients with pressure ulcers (stage III/IV) which were treated with rh-FGF2 showed better wound closure [34]. Another significant positive effect of pressure ulcers, in a study by Robson et al., indicated that wounds treated with rh-FGF2 had better healing rates than rh-GM-CSF (recombinant human granulocyte-colony stimulating factor) treatment alone [35].

The effect of rh-FGF2 on diabetic foot ulcers management showed a variable effect. In a randomized controlled trial, patients with diabetic ulcers on the plantar surface of the foot were randomized to receive topically applied rh-FGF2 or a placebo. The wounds were Wagner grade 1–3 and more than 0.5 cm at the largest diameter. A concentration of 5 μg/mL of rh-FGF2 or placebo was applied daily for 6 weeks and then twice a week for the following 12 weeks. After the study period, there was no difference observed between the healing rates for patients receiving rh-FGF2 or the placebo [40]. More recently, a randomized controlled clinical trial conducted by Uchi et al. of patients suffering from nonischemic diabetic ulcers with a Wagner grade of 2 and measuring 900 mm^2^ or less were randomized to placebo or treatment with 0.001% (50 μg) rh-FGF2 and 0.01% (500 μg) rh-FGF2 for up to 8 weeks. Ulcers treated with 0.01% rh-FGF2 showed a 75% or greater reduction in the area of the ulcer compared with the placebo group [41].

Treatment with FGF2 has shown a positive effect in the treatment of second-degree burns. In one randomized controlled trial, patients with superficial and deep second-degree burns were randomly selected to receive either a placebo or daily topical recombinant bovine rb-FGF2. All patients treated with rb-FGF2 exhibited faster granulation tissue formation and epidermal regeneration than those in the placebo group. Superficial and deep second degree burns treated with rb-FGF2 healed in 9.9 and 18.0 days on average, respectively, were significantly better than the placebo group [42]. Topical oxygen therapy supplementing FGF2 application accelerated deep second-degree burn healing [43]. A clinical research reported an effect of FGF2 for pediatric hand burns. In comparison to the control, FGF2 treatment was effective in cases that healed within 21 days, avoiding scar contractures and hypertrophic scars [44].

### 3.2. Prospect of FGF Applications in Endocrine Metabolic Regulation

Three FGF19 subfamily members, FGF19, FGF21, and FGF23, do not activate their cognate receptors in a heparan sulfate-dependent manner because of their poor affinity to heparan sulfate. Instead, they bind to Klotho to activate and dimerize their receptors. Although FGFRs are widely distributed, Klotho-dependent endocrine FGFs and Klotho expression are tissue-specific. Klotho is only expressed in the white adipose, pancreas, liver, testis, and kidney. The special ligand regulation of the FGF19 subfamily allows diverse biological properties of the FGF19 subfamily members but, more importantly, offers a new hope to the treatment of various human metabolic diseases [45,46,47].

Recent studies show that the FGF21 agonist is a promising therapeutic agent for the treatment of type 2 diabetes and obesity [48,49]. FGF21 is a polypeptide found in mouse embryonic cells, it is primarily secreted by the liver, and acts in endocrine regulation by binding to FGF receptors found on the cell surface. FGF21 plays an important role in the regulation of glucose and lipid metabolism in obese rodents and primates, acting to reduce blood sugar and triglyceride levels, enhancing sensitivity to insulin, and reducing body weight [50]. Interestingly, FGF21 injection does not cause hypoglycemia in rodents and primates, and studies have shown that FGF21 can act directly on the pancreas to regulate its function [50]. Immunostaining of the pancreatic islet shows that both the number of islets and insulin content were increased in FGF21-injected db/db mice [51,52,53]. In an animal study, we found the insulin-sensitizing adipokine adiponectin was a downstream effector of FGF21. FGF21 enhanced both expression and secretion of adiponectin in adipocytes, and adiponectin couples FGF21 actions in local adipocytes to the liver and skeletal muscle to mediate the systemic effects of FGF21 on energy metabolism and insulin sensitivity. Based on this, we concluded that FGF21 regulates glucose and lipid metabolism through the PLC-PPAR -adiponectin (APN) signaling pathway [54]. Extensive clinical and animal data indicate that FGF21 is a promising first-line treatment drug for type 2 diabetes. Eli Lilly reported the effects of LY2405319 (LY), a variant of FGF21, in a randomized, placebo-controlled, double-blind, proof-of-concept trial in patients with obesity and type 2 diabetes. Patients received a placebo or 3, 10, or 20 mg of LY daily for 28 days. LY treatment produced significant improvements in dyslipidemia, including decreases in low-density lipoprotein cholesterol and triglycerides and increases in high-density lipoprotein cholesterol and a shift to a potentially lower atherogenic apolipoprotein concentration profile. Favorable effects on body weight, fasting insulin, and adiponectin levels were also detected [55,56]. A study of LY2405319 in participants with type 2 diabetes (Phase 1) was completed in May 2013. A similar FGF21 product from Amgen is currently being developed at the pre-clinical stage [57].

FGF21 is also implicated in cardiovascular diseases. Patients with coronary artery disease exhibit significantly higher plasma FGF21 levels, positively associated with serum total cholesterol, TG, and HDL levels [58,59,60,61]. In an ex vivo Langendorff system, FGF21 treatment resulted in a cardioprotective effect and restored cardiac function via autocrine/paracrine pathways. However, these protective effects were reduced in obese mice [62]. In addition, FGF21-KO mice were more sensitive to isoproterenol-induced cardiac hypertrophy than wild-type mice, as characterized by increased heart weight, ventricular dilation, and cardiac dysfunction. These pathological changes were reversed by the administration of recombinant FGF21 [63].

More recently, our group elucidated the molecular mechanisms underlying the therapeutic effects of FGF21 in the treatment of atherosclerosis [54]. FGF 21 potently alleviates atherosclerotic plaque formation and decreases premature death in apolipoprotein E^–/–^ mice. First, FGF21 induces adiponectin production, which in turn directly acts on the blood vessels to inhibit neointima formation and macrophage inflammation. Second, FGF21 directs autocrine activities in the liver to suppress lipid biosynthesis, thereby reducing hypertriglyceridemia and hypercholesterolemia. Consistent with findings by Lin in mice, administration of an FGF21 analog in type 2 diabetic patients with obesity reduced several cardiovascular risk factors, including insulin resistance, dyslipidemia, and hypoadiponectinemia [56]. Notably, FGF21 is a downstream effector for both nuclear receptors, peroxisome proliferator-activated receptor (PPAR) α and γ, the agonists of which are effective for the treatment of both metabolic and vascular diseases [64]. Therefore, FGF21 or analogs may represent a promising cure for atherosclerotic diseases via multiple activities in adipose tissue, blood vessels, and liver.

FGF23 is associated with the occurrence of many phosphorus metabolism disorder diseases, such as inherited hypophosphatemic rickets, familial tumor-like calcification, X-linked hyperphosphatemia, and tumor-associated phosphorus osteomalacia. In addition, serum FGF23 concentration has been associated with the progression of chronic kidney disease [65,66]. Thus, FGF23 antagonists can be used to reduce inherited and tumor-induced hypophosphatemia, and organ transplantation and intravenous iron treatment-caused hypophosphatemia. Amgen has studied the effect of FGF23 monoclonal antibody on chronic kidney disease-mineral and bone disorder (CKD-MBD) using a rat model. They evaluated the impact of FGF23 neutralization using an antibody to target CKD-MBD, secondary hyperparathyroidism (HPT), and relevant concurrent diseases. CKD-MBD rat models fed a high-phosphorus diet were treated with either a high dose or a low dose of monoclonal FGF23 antibody. The results showed that treatment with the monoclonal FGF23 antibody inhibited the occurrence of secondary HPT and led to decreased parathyroid hormone levels, increased vitamin D and calcium levels, and restored bone markers such as cancellous bone and bone mass back to normal levels [67]. Kyowa Hakko Kirin (KHK) of Japan collaborated with Ultragenyx to develop humanized recombinant human FGF23 monoclonal antibody, KRN23, for the treatment of X-linked hypophosphatemia (XLH), a rare metabolic bone disorder. At present, KHK is completing phase I/II clinical trials of KRN23 in adult XLH patients in the US and Canada [68]. KRN23 antagonizes and inactivates circulating FGF23, thereby enhancing renal tubular absorption of urine phosphate and increasing the levels of phosphorus in the blood [69]. Studies of the pharmacokinetics (PK) and pharmacodynamics (PD) of KRN23 in the first multiple ascending-dose trial treating adults with XLH showed that the PK dose proportionality and the linear PK/PD relationship between serum KRN23 and serum inorganic phosphorus (Pi) concentrations support a treatment strategy to adjust the KRN23 dose based on serum Pi levels [70]. KRN23 is expected to become the first XLH-specific therapeutic drug to target FGF23 overexpression in XLH patients.

### 3.3. Advances in FGFR Inhibitors with Antitumor Activity

Aberrant FGFR expression or gene mutations can contribute to the occurrence and development of various tumors [71,72,73,74,75]. Various studies have shown that the FGFR1 gene is over-amplified in lung cancer and estrogen receptor-positive breast cancer [76,77,78], FGFR2 overexpression is related to diffuse gastric cancer and triple-negative breast cancer [79,80,81], FGFR1 chromosomal translocation occurs at the 8p11 locus in myelodysplastic syndrome and alveolar rhabdomyosarcoma [82], FGFR3 chromosomal translocation occurs in multiple myeloma and peripheral T-cell lymphoma [83,84], missense mutations in the FGFR2 gene are found in endometrial cancer and melanoma [85,86], mutations in the FGFR3 gene result in invasive bladder cancer [87,88], and the FGFR4 mutation is correlated with Rhabdomyosarcoma [89,90]. Therefore, FGFR inhibitors are a new focus in the field of cancer therapy. A variety of small molecule FGFR inhibitors target FGFR gene mutations and overexpression, including dovitinib, KI23057, and AZD4547, and have shown significant therapeutic effects in pre-clinical and clinical studies [22]. In addition to small molecule inhibitors, FGFR-targeting monoclonal antibodies have demonstrated dramatic anti-tumor activities in tumor cell lines and animal models. For example, km1334, a FGF8b-targeting neutralizing antibody, blocks FGF ligand-mediated signaling pathways in breast cancer. This antibody also has substantial therapeutic effects in FGF8b-overexpression prostate cancer with inflammation and bone destruction [91]. Gp369 is a FGFR2 IIIb-specific blocking antibody that inhibits the proliferation of a variety of human cancer cells and xenograft tumors with FGFR2 gene amplification and point mutations (S252W, n550k) [79,92,93,94]. R3mab, a FGFR3 (IIIB and IIIc isoforms)-specific monoclonal antibody (does not affect FGFR1, FGFR2, or FGFR4), has been shown to significantly inhibit FGF1-induced cancer cell proliferation [95].

Another new therapeutic strategy is called an FGF “ligand trap” and is comprised of a fusion protein of an immunoglobulin Fc fragment and a soluble FGFR extracellular domain that competitively binds to FGF1, 2, 3, 7 and 10 to inhibit ligand-dependent FGFR signaling [96]. One example of this is Fp-1039, an experimental cancer therapeutic drug developed as a “ligand trap” with an FGFR1c ligand-binding domain and a human immunoglobulin Fc fragment [97,98]. Fp-1039 has a dramatic inhibitory effect on head and neck squamous cell carcinoma overexpressing FGF2, but has no effect on squamous cell carcinoma with low FGF2 expression [22]. Fp-1039 is currently in phase II clinical trials to treat endometrial carcinoma. Although FGFR inhibitors have shown potent anti-tumor activities, it is imperative to identify specific tissues that are susceptible to the toxicity of FGFR inhibitors and also determine the appropriate therapeutic doses of the inhibitors, since FGFRs are widely expressed in many tissues and play important roles in human body development and physiology. To that end, identification of the most efficacious FGFR isoform inhibitor with a potent therapeutic effect but only minimal side effects would be the most ideal outcome. In summary, FGFR inhibitors act through multiple mechanisms and may overcome drug resistance of tumors, providing a novel strategy for the treatment of refractory tumors.

### 3.4. Advances in Basic and Applied FGF Research in China

Chinese scientists are at the forefront of efforts to develop FGF drugs. In 1998, Lancet magazine reported the clinical studies performed by Chinese scientists to test FGF2 in the treatment of severe burns in 600 patients. The results showed that FGF2 significantly reduced the healing time and improved the quality of wound healing [99]. In 2000, recombinant human basic fibroblast growth factor (rb-FGF2) was approved for use by the Chinese Food and Drug Administration for the treatment of chronic wounds including chronic granulating wounds, ulcers and bedsores, fresh wounds (trauma and surgical wounds) and burn wounds (superficial degree burn, deep second degree burns, and granulation wounds). Subsequently, FGF2 has been formulated as gels, sprays, eye drops, and other formulations [100,101]. Lin T et al. reported the application of FGF2 eye drops for the treatment of mechanical damage-induced superficial corneal abrasions and the results indicated that 3-day application of FGF2 gave an overall therapeutic efficiency of 93.33%, higher than the 86.67% for sodium hyaluronate (control). After 7 days of treatment, the corneal damage area in the FGF2 treatment group was reduced from 10.58 mm^2^ to 0.02 ± 0.07 mm^2^ [100]. Therapeutic effects of rb-FGF2 were also observed in the foot of patients with early diabetes by Wang, Z.H., et al. [102]. Specifically, the healing rate and inflammatory exudates in the rb-FGF2 group were significantly better than those in the vaseline gauze (control) group (*p* < 0.05) 2 weeks after application.

In 2006, recombinant human acidic fibroblast growth factor (rh-FGF1) was marketed in China primarily for the treatment of deep second degree burn wounds and chronic ulcers including flat residual traumatic wounds, diabetic ulcers, vascular ulcers, and bedsores. To investigate the efficacy of topical recombinant human acidic fibroblast growth factor (rh-FGF1) treatment in deep partial-thickness burn or skin graft donor sites, a randomized, multicenter, double-blind, and placebo-controlled clinical trial was conducted at the Department of Burn Surgery, Changhai Hospital, the Second Military Medical University, Department of Burn Surgery, Affiliated with the First Hospital of the PLA general Hospital and Department of Burn Surgery, Jishuitan Hospital [103]. The healing rate, fully healed rate, and healing time were evaluated to assess the efficacy of rh-FGF1 application. The results showed that the healing rate of burn wounds and skin graft donor sites treated by rh-FGF1 was significantly higher than that by the placebo, and the mean healed time of burn wounds and skin graft donor sites in the rh-FGF1 group were significantly shorter than that in the placebo group. Wu, J.C. et al. reported that use of FGF1 for spinal cord injury was safe and significant improvements in ASIA motor and sensory scale scores, ASIA impairment scales, neurological levels, and functional independence measure at 24 months after treatment were noted [104].

In 1996, the world’s first recombinant bovine basic fibroblast growth factor (rb-FGF2) was released in China. In 2005, rh-FGF1 was developed by Chinese scientists and was approved by the Chinese Food and Drug Administration as the world's first listed FGF1 drug. The FGF2-containing collagen sponge polymeric material developed by Chinese scientists is presently the only approved FGF-containing biomaterial. These research achievements effectively stimulate FGF drug development all over the world. In Science magazine, US scientist Sun praised Chinese scientists for their contributions to the development of new FGF drugs, and emphasized the importance of the newly approved FGF2 drug developed in China, developed to treat angiogenesis, granulation tissue formation, and hair follicle regeneration during wound healing [105].

Fifteen years of clinical studies in wound healing have shown that FGF is effective and safe for use to stimulate wound repair, accelerate healing, and reduce both scarring and pigmentation. In fact, novel FGF-based drugs are included on the Chinese National Reimbursement Drug List, including rb-FGF2 eye ophthalmic gel, rb-FGF2/rh-FGF2 eye drops, lyophilized rb-FGF2/rh-FGF2 for topical use, and rh-FGF2-containing collagen sponge polymeric material.

## 4. Conclusions

Thirty years have passed since the identification of the first FGF cDNA sequence. Numerous studies have shown that FGF signaling participates in almost all processes of development, physiology and pathology. FGF/FGFR signaling acts in processes from the early stage of embryonic development to organogenesis, tissue maturation, tissue homeostasis, damage repair, and the development of cancer. The discovery of endocrine FGFs revealed new mechanisms underlying the FGF regulation of glucose and lipid metabolism and mineral balance, providing new therapeutic targets for the treatment of type 2 diabetes, chronic kidney disease, and obesity. The contribution of FGF/FGFR gene amplification, mutation, and integration to the pathophysiology of chronic diseases such as rickets, craniosynostosis, and various cancers, provides additional therapeutic strategies for these diseases. Further research on the pharmacology and toxicology of highly selective FGF agonists and antagonists will facilitate new therapeutic approaches for tissue repair, metabolic diseases, and cancers. More in-depth studies of FGF structure, signaling, and regulatory mechanisms should further improve the utility and expand applications of FGF-based therapeutics.
